# Supplementary material for: Validation of physical activity levels from shank-placed Axivity AX6 accelerometers in older adults
Source: PLoS One. 2024 May 13;19(5):e0290912. doi: 10.1371/journal.pone.0290912 (PMC11090333; doi:10.1371/journal.pone.0290912)
Supplement: S1 Table — Axivity Data is missing from Participant 21 due to early removal of the Axivity AX6 sensor. (DOCX) [file pone.0290912.s001.docx]

|  | | | **Axivity % of Time in Intensity (LOOCV)** | | | | **ActiGraph % of Time in Intensity** | | | |
| --- | --- | --- | --- | --- | --- | --- | --- | --- | --- | --- |
| **Participant ID** | **Optimized Cut-point** | **LOOCV Cut-point** | **SB** | **LPA** | **MPA** | **VPA** | **SB** | **LPA** | **MPA** | **VPA** |
| PA_01 | 289, 10384, 36480 | 792, 8356, 18194 | 80.9 | 13.9 | 1.1 | 4.2 | 76.4 | 19 | 4.6 | 0 |
| PA_02 | 476, 9360, 16000 | 787, 8387, 18814 | 85.1 | 13.7 | 1.2 | 0 | 82.3 | 16.6 | 1.1 | 0 |
| PA_03 | 511, 6192, 16000 | 786, 8483, 18814 | 71.2 | 27.8 | 1.0 | 0 | 67.9 | 29.4 | 2.7 | 0 |
| PA_04 | 442, 7952, 24752 | 788, 8430, 18549 | 73.1 | 20.6 | 1.8 | 4.5 | 68.9 | 24.4 | 2.2 | 4.4 |
| PA_05 | 612, 7024, 22656 | 783, 8458, 18612 | 69.3 | 27.9 | 2.3 | 0.4 | 67.4 | 29.2 | 3.2 | 0.1 |
| PA_06 | 720, 6032, 16000 | 779, 8488, 18814 | 88.7 | 11.3 | 0 | 0 | 88.1 | 11.7 | 0.2 | 0 |
| PA_07 | 209, 10288, 16000 | 795, 8359, 18814 | 79.3 | 17.9 | 2.7 | 0 | 73.0 | 25.6 | 1.4 | 0 |
| PA_08 | 572, 7952, 15264 | 784, 8430, 18836 | 79.7 | 18.9 | 1.4 | 0 | 77.6 | 20.9 | 1.4 | 0.2 |
| PA_09 | 825, 10512, 16000 | 776, 8352, 18814 | 84.8 | 14.9 | 0.3 | 0 | 85.3 | 14.6 | 0.2 | 0 |
| PA_10 | 804, 7568, 20096 | 777, 8441, 18690 | 83.2 | 14.2 | 1.9 | 0.7 | 83.5 | 13.7 | 2.2 | 0.6 |
| PA_11 | 988, 8464, 16000 | 771, 8414, 18814 | 80.1 | 18.5 | 1.4 | 0 | 82.0 | 16.6 | 1.4 | 0 |
| PA_12 | 812, 6400, 16000 | 777, 8477, 18814 | 78.7 | 19.9 | 1.4 | 0 | 79.0 | 18.9 | 2.1 | 0 |
| PA_13 | 748, 16144, 16244 | 778, 8182, 18807 | 78.0 | 19.7 | 1.3 | 1 | 77.6 | 21.0 | 1.3 | 0 |
| PA_14 | 1081, 7568, 16000 | 768, 8441, 18814 | 83.6 | 16.0 | 0.4 | 0 | 86.0 | 13.2 | 0.8 | 0 |
| PA_15 | 1220, 6096, 16000 | 764, 8486, 18814 | 89.4 | 10.1 | 0.5 | 0 | 92.3 | 7.0 | 0.7 | 0 |
| PA_16 | 555, 8464, 16000 | 784, 8414, 18814 | 79.9 | 17.7 | 2.4 | 0 | 78.1 | 19.6 | 2.3 | 0 |
| PA_17 | 407, 6384, 32384 | 789, 8477, 18318 | 76.3 | 21.6 | 1.6 | 0.5 | 72.1 | 25.5 | 2.4 | 0 |
| PA_18 | 1124, 10000, 16000 | 767, 8368, 18814 | 84.2 | 14.1 | 1.7 | 0 | 86.0 | 12.5 | 1.5 | 0 |
| PA_19 | 952, 10704, 16000 | 772, 8346, 18814 | 78.4 | 19.8 | 1.9 | 0 | 80.3 | 19.3 | 0.5 | 0 |
| PA_20 | 984, 9584, 16000 | 771, 8380, 18814 | 75.9 | 21.9 | 2.2 | 0 | 78.8 | 20.2 | 1.0 | 0 |
| PA_21 |  |  |  |  |  |  | 84.7 | 15.2 | 0.2 | 0 |
| PA_22 | 1500, 8976, 16000 | 756, 8399, 18814 | 76.8 | 23.0 | 0.2 | 0 | 84.7 | 15.2 | 0.1 | 0 |
| PA_23 | 1076, 10248, 16000 | 769, 8360, 18814 | 76.5 | 20.8 | 2.7 | 0 | 79.2 | 19.0 | 1.8 | 0 |
| PA_24 | 412, 10768, 32384 | 789, 8344, 18318 | 85.5 | 12.8 | 1.7 | 0 | 81.9 | 16.8 | 1.3 | 0 |
| PA_25 | 888, 8848, 20096 | 774, 8403, 18690 | 70.4 | 24.0 | 3.4 | 2.2 | 71.6 | 22.9 | 3.2 | 2.2 |
| PA_26 | 676, 8720, 36480 | 781, 8407, 18194 | 81.0 | 15.6 | 3.2 | 0.3 | 80.0 | 16.5 | 3.5 | 0 |
| PA_27 | 788, 7184, 14080 | 777, 8453, 18872 | 78.4 | 19.9 | 1.7 | 0 | 78.6 | 19.4 | 1.3 | 0.7 |
| PA_28 | 580, 7952, 16000 | 784, 8430, 18814 | 89.3 | 10.7 | 0.1 | 0 | 87.5 | 12.5 | 0.1 | 0 |
| PA_29 | 709.5, 7440, 16000 | 780, 8445, 18814 | 77.4 | 22.4 | 0.2 | 0 | 76.6 | 23.0 | 0.4 | 0 |
| PA_30 | 1310, 7296, 16000 | 761, 8450, 18814 | 72.0 | 27.2 | 0.7 | 0 | 78.3 | 20.7 | 1 | 0 |
| PA_31 | 900, 6928, 16000 | 774, 8461, 18814 | 80.1 | 17.7 | 2.2 | 0 | 81.4 | 16.0 | 2.6 | 0 |
| PA_32 | 716, 10000, 16000 | 779, 8368, 18814 | 79.3 | 20.7 | 0 | 0 | 78.5 | 21.5 | 0 | 0 |
| PA_33 | 918, 7696, 16000 | 773, 8438, 18814 | 79.8 | 20.1 | 0.1 | 0 | 80.9 | 18.9 | 0.1 | 0 |
| PA_34 | 572, 4368, 16000 | 784, 8538, 18814 | 89.8 | 10.2 | 0 | 0 | 86.8 | 13.0 | 0.2 | 0 |
| PA_35 | 1044, 6640, 13952 | 769, 8470, 18876 | 77.9 | 21.6 | 0.4 | 0 | 81 | 18.1 | 0.9 | 0 |
