# Supplementary material for: Validation of physical activity levels from shank-placed Axivity AX6 accelerometers in older adults
Source: PLoS One. 2024 May 13;19(5):e0290912. doi: 10.1371/journal.pone.0290912 (PMC11090333; doi:10.1371/journal.pone.0290912)
Supplement: Attachment — Submitted filename: Review.docx [file pone.0290912.s002.docx]

# Review – Plos One - PONE-D-23-25684

## Reviewer’s comments

The paper analyzed shank-mounted Axivity data to classify the physical activity levels on older adults during recordings lasting 72 hours. Congratulations to the authors, the study is interesting. In general, the paper has a high-quality English language and the results are clearly presented, but further analyses and comments should be added and discussed.

Title

I suggest to stress the fact that the population include older adults.

### Introduction

1. Please describe more in details the methods and findings of similar works focusing on the classification of physical activity levels (e.g., device locations, selected cut-points, methods to select them, etc.).
2. Please briefly define the activity characteristics mainly related to sedentary behavior, light, moderate and vigorous physical activity levels.

### Methods

#### Procedures

Please cite a reference paper dealing with the choice of attaching the Actigraph above the right iliac crest (and not at L5 for instance) and explain the main reasons.

#### Data Analysis

1. I suggest to explicitly mention the Freedson cut-points values.
2. The authors sequentially optimized the cut-points from the sedentary behavior till the vigorous physical activity. Did you try to invert the order of the physical activity levels optimization? Do you think that the results would change?
3. The authors performed the individual-based optimization process to minimize the differences between the hip-worn Actigraph and the shank-worn Axivity classification, then performed a leave-one-out cross-validation using 34 averaged individual-based cut-points on the left 35^th^ subject. Then did you use a single set of cut-points (the best set selected with the cross-validation) on a test set? It would be crucial to apply fixed cut-points on different data or at least on the whole dataset to assess the classification performances.

### Results

1. I suggest to move the comments on excellent/good agreement or reliability to Discussion.
2. Do you show the classification results against ActiGraph adopting the resultant cut-points reported in Table 4?

### Discussion

1. Please avoid repetitions such as the lines 265-266.
2. Please better justify or hypothesize why the cut-point for vigorous physical activity level at ankle found by Rhudy et al. is lower than the cut-point at shank found in this study. Healthy young populations, as the one enrolled by Rhudy et al., does not seem to show lower ankle accelerations than shank accelerations of older adults.

Conclusion

Since the sampling frequency and the acceleration amplitude affect the results, I suggest to stress that the proposed cut-points for physical activity are estimated on older adults and using accelerometers with sampling frequency equal to 100 Hz.

Data availability

Is it possible to share the raw data from ActiGraph and Axivity?
