# Supplementary material for: Validation of physical activity levels from shank-placed Axivity AX6 accelerometers in older adults
Source: PLoS One. 2024 May 13;19(5):e0290912. doi: 10.1371/journal.pone.0290912 (PMC11090333; doi:10.1371/journal.pone.0290912)
Supplement: Attachment — Submitted filename: Response to Reviewers.docx [file pone.0290912.s003.docx]

**Reviewer 1 Comments:**

Thank you for your thoughtful and thorough comments in reviewing our manuscript. We have addressed all your concerns, and we feel the manuscript is much improved. The notes below highlight these changes and hope they make it easy to follow these revisions.

1. **You talked a lot about PA, but which kind of PA were the subjects asked to perform?**

Response: Thank you for highlighting this point of unclarity. They were not asked to perform specific amounts of any intensity of physical activity. To your point, while some researchers may instruct participants to perform varying levels of PA, such as Rhudy M.B., et al. 2020, we chose to study free-living PA, similar to a study done by Dutta A. et. al., 2018. This has also been clarified on lines 143-144. Participants were asked to perform their free-living PA, meaning how much activity they undergo on a routine basis.

- Dutta, A., Ma, O., Toledo, M., Pregonero, A. F., Ainsworth, B. E., Buman, M. P., & Bliss, D. W. (2018). Identifying Free-Living Physical Activities Using Lab-Based Models with Wearable Accelerometers. Sensors (Basel, Switzerland), 18(11), 3893. <https://doi.org/10.3390/s18113893>

2. **Line 55: "objective measures of PA" such as? I would suggest to mention some of them.**

Response: Excellent point, this has now been updated to include objective measures of PA on lines 55-56. The objective measures listed include step counts, time spent in different intensity levels, duration of activity, and energy expenditure (Silfee V.J. et. al., 2018)

Silfee VJ, Haughton CF, Jake-Schoffman DE, Lopez-Cepero A, May CN, Sreedhara M, et al. Objective measurement of physical activity outcomes in lifestyle interventions among adults: A systematic review. Prev Med Rep. 2018;11: 74–80. doi:10.1016/j.pmedr.2018.05.003

3. **Line 64: "activity counts", what are they? How are they identified? Based on which criterion?**

Response: Thank you for identifying this weakness in our previous draft. Activity counts are numerical values which reflect the intensity and frequency of movement over a specified time period, usually per minute. Activity that causes the acceleration signal from the sensor to exceed a threshold is essentially ‘counted’ as activity, whereas those below the threshold are not counted. Typically, higher activity counts means moderate and vigorous physical activity is being performed, whereas lower activity counts constitute sedentary and light physical activity. In terms of which criterion they are identified, this is based on thresholds and cut-points which are used to classify them as the different types of PA and connects well to the purpose of the study, of aiming to determine these cut-points. We have added additional text that provide additional context on the topic of activity counts on lines 66-72.

4. **Line 66: it is stated that cut-points are used to classify the intensity, but firstly it would be appreciated if it is explained what these cut-points are and then how they are identified (trial and error or mathematically or what so ever). It is a key point for good comprehension of the work.**

Response: Cut-points are the thresholds in the activity count spectrum which serve as reference points to categorize the four PA intensity levels (SB, LPA, MPA, and, VPA). These cut-points are based on 60-second epoch lengths and they are specific to the wear location of the device. They are determined through validation and calibration studies, where researchers often ask participants to engage in activities of known intensity. By comparing the recorded activity counts with expected intensity levels, researchers can then establish cut-points that differentiate between the four PA levels. Based on your feedback, we have also further explained the topic of cut-points on lines 76-84.

5. **Line 111: in this paragraph it is reported the number of used sensors and their location, but a figure should be included to better understand the considered experimental protocol.**

Response: Yes, thank you for the idea. A figure has now been added to the manuscript. This figure is shown below, with the caption located at line 176 in the text.

Fig 1. Wear locations of the ActiGraph and the Axivity for each participant

6. **Line 124-131: ActiGraph and Axivity are two different types of sensors that were used, the first one as the gold standard and the second one to be validated. The ActiLife software was used to acquire simultaneously the two sensors, so was the signal to start the recording given directly by the software to both type of sensors?**

Thank you for highlighting this point of confusion. The ActiGraph was initialized through the ActiLife software but was time-synchronized with the computer. Based on this, we configured the settings of the ActiGraph to start recording at a set time. The Axivity was initialized through the OpenMovement GUI, which was also time-synchronized with the same computer. It was also configured by us to start recording at the same time that was chosen for the ActiGraph. This was so that we would record the same activities performed by the participant between the two sensors. The two devices themselves were not synchronized to each other. This is now clarified on lines 154-157.

7. **Moreover, nothing is said about the preprocessing of the data, such as filtering, do this mean that no pre-processing has been done and data has been used in their raw form or something different? I suggest to report the preprocessing part if done, if not performed to specify it and justify why.**

You are correct in assuming that raw data was used for this study. This is noted on line 185 which states “Raw data from the Axivity sensor were downloaded through the OpenMovement...”. For the ActiGraph data, this is post-processed using the ActiLife software, which has limited information available as to how it is processed, due to proprietary reasons. For the Axivity data, pre-processing such as filtering was also not done, but was completed at the post-processing stage to ensure optimal signal quality, noise reduction, and alignment with the study’s analytical objectives. Overall, given our limited knowledge of the ActiGraph's functions, our intention is to preserve as much of the original signal as we can.

8. **Line 140-144: to make a fairer comparison you should verify that the results given by this MATLAB script are the same of those given by the ActiLife software using the same data, i.e. those of the ActiGraph (if it has been already done, please specify and add reference if necessary).**

Thank you for raising this important point. It must be noted that this script was chosen carefully because it follows the published methods to replicate ActiGraph equivalent activity counts (Brønd JC, 2017). This study found a high degree of agreement in the PA intensity classification as the ActiGraph and associated ActiLife software, when using raw accelerometer data. So far, this has also been cited by 51 other articles in the PA field. While other measures (e.g., step count, step length) have not been validated, this particular activity count script has been validated. Also, as elaborated on in the discussion from lines 331 to 352, we compared our results of activity counts and cut points from other wear locations on the lower limb. This analysis suggested that our estimates of activity counts are in line with previous studies.

9. **Line 148: you stated that an optimization algorithm was created and a short description follows; you should go more in depth in this part, it is not enough, a more accurate description of the optimization algorithm should be provided, which is the reasoning behind and the chosen criteria.**

Thank you for highlighting this point of confusion. This has now been elaborated on from lines 207 to 214.

10. **Line 151: are the data collected prior to this study referred to the same type of PA? In my opinion, they should be the same type of PA to be included as starting point.**

It is now clarified that this is free-living PA on line 200. These collections were shorter in length, in which data was only recorded for 48 hours compared to the 72 in the study, and had fewer collections (n=4) than those listed in this study, to give us a starting point for our algorithm. These details have been added in the manuscript on lines 201 to 203.

11. **Line 249: "the time spent in..." the sentence is not concluded, please conclude it.**

Thank you, this has now been done.

12. **Regarding the participants, it is not clear if the participants are all healthy or all pathological or a mix of them. As it is written, it seems that it is not a homogeneous set of subjects, in my opinion mixing data from healthy and pathological subjects (moreover different among them in the pathology) is not a fair, since this could influence the results. Could you specify better the healthy status of the subjects?**

Thank you for commenting on this. Essentially, they were all older adults that did not reside in long-term care homes and did not require any walking aids or had any major neurological conditions that would affect their walking. We also included participants that did have diagnosed osteoarthritis, some which had osteoarthritis of the knee. Based on their mean Oxford Knee Score shown in Table 1, which was 37.3, it is on the closer end to being fully functional and only indicates mild to moderate knee osteoarthritis, meaning that their knee osteoarthritis had minimal impact on their ability to perform physical activity. The reason why we included adults with knee osteoarthritis or previous lower limb surgeries and replacements was because we wanted our data to be as generalizable to the older adult population as possible. This has also now been clarified in the participants section in lines 126, and 129-133.

13. **About the location of the sensor that you wanted to validate, it is clear that it is an ideal position for free-gait analysis but it is neither a comfortable position for the subject nor a location that could be included for instance in the clothes, so why should be used?**

Response: The selected sensor location, while optimal for capturing shank accelerations for gait analysis, does have similar potential challenges related to subject comfort and practical wearability that commercial devices, such as smart watches have. This is especially true because it may not be easily integrated into everyday clothing. Despite these considerations, the shank remains a widely researched location to provide valuable insights that can be extended to classify the health of individuals impacted by injuries and diseases affecting the knee, such as knee osteoarthritis patients. Therefore, the purpose of this wear location would be to provide additional insights, such as PA data, to researchers that are placing sensors at this location. Being able to obtain PA data from a single sensor like this would reduce the need to have the patient wear a second sensor, which would imrpvoe the overall patient experience, and likely compliance. Studies done by Li. Q. et al., 2010, Celik Y. et al., 2021, which are cited in the paper, as well as some studies done by Kobsar D., 2020 which are listed below, support the shank as a common wear location in gait analysis. Furthermore, this wear location, specifically at the flat part of the shin has been studied for reliability by Ruder M. 2023.

- Kobsar, D., Charlton, J. M., Tse, C. T. F., Esculier, J. F., Graffos, A., Krowchuk, N. M., Thatcher, D., & Hunt, M. A. (2020). Validity and reliability of wearable inertial sensors in healthy adult walking: a systematic review and meta-analysis. Journal of neuroengineering and rehabilitation, 17(1), 62. <https://doi.org/10.1186/s12984-020-00685-3>
- Kobsar D, Masood Z, Khan H, Khalil N, Kiwan MY, Ridd S, Tobis M. Wearable Inertial Sensors for Gait Analysis in Adults with Osteoarthritis—A Scoping Review. Sensors. 2020; 20(24):7143. <https://doi.org/10.3390/s20247143>
- Ruder, M. C., Masood, Z., & Kobsar, D. (2023). Reliability of waveforms and gait metrics from multiple outdoor wearable inertial sensors collections in adults with knee osteoarthritis. Journal of biomechanics, 160, 111818. Advance online publication. <https://doi.org/10.1016/j.jbiomech.2023.111818>

**Reviewer 2 Comments:**

Thank you for your thorough review of our manuscript. Your insightful comments and questions have helped us refine our work. We have carefully addressed each of your concerns and we believe our manuscript is much improved as a result of these comments and revisions. The notes below highlight these changes and hope they make it easy to follow these revisions.

Title

1. **I suggest to stress the fact that the population include older adults.**

Thank you, we have updated the title to specify that our study population consists of older adults.

Introduction

2. **Please describe more in details the methods and findings of similar works focusing on the classification of physical activity levels (e.g., device locations, selected cut-points, methods to select them, etc.).**

These details have been elaborated on from lines 79 to 84, where this further discussion on how cut-points are determined from other studies. A study that I believe is a prime example of how physical activity is classified with accelerometers is by AHK Montoye et. al., (2020), where it aimed to develop cut-points from a wrist-worn ActiGraph in free-living adults, by comparing the activity count distributions for different activity types of known intensities.

- *Montoye AHK, Clevenger KA, Pfeiffer KA, Nelson MB, Bock JM, Imboden MT, et al. Development of cut-points for determining activity intensity from a wrist-worn ActiGraph accelerometer in free-living adults. J Sports Sci. 2020;38: 2569–2578. doi:10.1080/02640414.2020.1794244*

3. **Please briefly define the activity characteristics mainly related to sedentary behavior, light, moderate and vigorous physical activity levels.**

Thank you, for this comment. Examples of these varying activity levels have now been provided from lines 72-76 within the introduction section.

Methods

*Procedures*

4. **Please cite a reference paper dealing with the choice of attaching the Actigraph above the right iliac crest (and not at L5 for instance) and explain the main reasons.**

This is a great point you make. The choice of wear location was clarified in lines 146-150. Essentially, this wear location comes from the Freedson et. al. (1998) paper which studies Actigraphy when the device is placed above the right hip. While the right hip is a general location, for consistency I instructed just above the right iliac crest for each participant. The right iliac crest is also an anatomical landmark which participants can find easily in case the ActiGraph device shifts during activities. This wear location for the ActiGraph has also been used by numerous other studies that also utilize the Freedson cut-points, which include AHK Montoye et. al., (2020) and AM Leinonen et. al., (2017).

- *Montoye AHK, Clevenger KA, Pfeiffer KA, Nelson MB, Bock JM, Imboden MT, et al. Development of cut-points for determining activity intensity from a wrist-worn ActiGraph accelerometer in free-living adults. J Sports Sci. 2020;38: 2569–2578. doi:10.1080/02640414.2020.1794244*
- *Leinonen, A. M., Ahola, R., Kulmala, J., Hakonen, H., Vähä-Ypyä, H., Herzig, K. H., Auvinen, J., Keinänen-Kiukaanniemi, S., Sievänen, H., Tammelin, T. H., Korpelainen, R., & Jämsä, T. (2017). Measuring Physical Activity in Free-Living Conditions-Comparison of Three Accelerometry-Based Methods. Frontiers in physiology, 7, 681.* [*https://doi.org/10.3389/fphys.2016.00681*](https://doi.org/10.3389/fphys.2016.00681)

*Data Analysis*

5. **I suggest to explicitly mention the Freedson cut-points values.**

Thank you, these have now been stated on lines 181-182 and have been cited as well.

6. **The authors sequentially optimized the cut-points from the sedentary behavior till the vigorous physical activity. Did you try to invert the order of the physical activity levels optimization? Do you think that the results would change?**

This has been theorized and tried; however it did not yield reasonable cut-points and changed the results greatly. This is because for many of the older adults in the population, their VPA levels were 0% according to the ActiGraph. Because an infinite amount of VPA cut-point values can yield 0% activity, the algorithm would choose the first cut-point value that would yield a 0% value, instead of fine-tuning the value after the MPA values.

7. **The authors performed the individual-based optimization process to minimize the differences between the hip-worn Actigraph and the shank-worn Axivity classification, then performed a leave-one-out cross-validation using 34 averaged individual-based cut-points on the left 35^th^ subject. Then did you use a single set of cut-points (the best set selected with the cross-validation) on a test set? It would be crucial to apply fixed cut-points on different data or at least on the whole dataset to assess the classification performances.**

Thank you for this question. A leave-one-out cross validation was done for all participants, not just the last 35^th^ participant. This allowed for testing of the cut-points on each participant separately, and the performance was assessed across the entire dataset.

This method of analysis is common in machine learning to ensure that the model or cut-points generalize well across different individuals rather than being tailored to a specific subset of the data. By using a leave-one-out cross-validation, each participant in the study is temporarily held out as a test set while the model or cut-points are trained on the remaining subjects. This process is repeated for each subject, and the overall performance is then assessed across the entire dataset.

In this sense, whilst following a leave-one-out cross validation model, testing on a different data set, continuing to use fixed cut-points on different data or the entire dataset may not be deemed necessary, especially as the focus is on ensuring the method generalizes well to diverse subjects, which is achieved through the testing done on each participant in the cross-validation process. This has also now been clarified in the text through lines 220-226.

Results

8. **I suggest to move the comments on excellent/good agreement or reliability to Discussion.**

While I do see your point of concern, I feel that the comments on excellent/good agreement or reliability are fitting in the conclusion, as these are labels based on the ICC values that were computed. To clarify this, the exact ICC values have also been added. As stated in the methods section (lines 234-236), the words excellent, good, and moderate were simply labels for the interpretation of the ICC values and do not include any of our own subjective interpretations of the results.

9. Do you show the classification results against ActiGraph adopting the resultant cut-points reported in Table 4?

Yes, Table 4 essentially depicts the cut-points that were found from our current study for the four PA intensity levels. ActiGraph currently does not have any current cut-points released for this wear location, hence the current study. These cut-points could be used with the ActiGraph, or really any accelerometer device, if their device is placed at the shank.

Discussion

10. Please avoid repetitions such as the lines 265-266.

Absolutely, these lines have been edited to reduce the repetition between the lines.

11. Please better justify or hypothesize why the cut-point for vigorous physical activity level at ankle found by Rhudy et al. is lower than the cut-point at shank found in this study. Healthy young populations, as the one enrolled by Rhudy et al., does not seem to show lower ankle accelerations than shank accelerations of older adults.

Thank you for highlighting this, the discussion has now been expanded on to include further reasoning and justification as to why we may see this in the dataset, which can be seen from lines 352 – 356.

Conclusion

12. Since the sampling frequency and the acceleration amplitude affect the results, I suggest to stress that the proposed cut-points for physical activity are estimated on older adults and using accelerometers with sampling frequency equal to 100 Hz.

Based on the algorithm used to process Axivity data into activity counts, the data is down sampled to 30 Hz, therefore sampling rate, while is important, is less important to sample at 100 Hz. 100 Hz was chosen at the sampling rate to mimic sampling at a rate that gait researchers do when they use wearable accelerometers on their subjects. However, the range is important to note to ensure accelerations being captured adequately, therefore this is included now in the conclusion section.

Data availability

13. Is it possible to share the raw data from ActiGraph and Axivity?

Unfortunately we do not have ethics to share raw data for this study. We agree that this is an important aspect of replicating research and have built open access data sharing into all projects upfront going forward.
