# Supplementary material for: Validation of physical activity levels from shank-placed Axivity AX6 accelerometers in older adults
Source: PLoS One. 2024 May 13;19(5):e0290912. doi: 10.1371/journal.pone.0290912 (PMC11090333; doi:10.1371/journal.pone.0290912)
Supplement: Attachment — Submitted filename: Response to Reviewers.docx [file pone.0290912.s004.docx]

Response to Reviewers

February 28, 2024

Reviewer #1:

Thank you for taking the time to review our paper. We truly appreciate the comments made and have addressed each one below. In doing so, we again believe this has further improved the quality of our paper.

1. **I would suggest just to add a table summarizing the acronyms of the different PAs, some examples of them (as already written in the text) and the corresponding cut-points found by the author.**

Thank you for this comment. We agree that this will be an important table to summarize the information from the study for readings. This information has been added to Table 4, which previously only communicated the resultant cut-points. Now, this table reflects the intensity in full, which also displays the abbreviations in the parentheses, and displays the examples of activity for each intensity, as shown below.

**Table 4. Resultant Shank Cut-points with Examples of Activity Shown for Each Intensity.**

|  |  | **Counts per Minute** | |
| --- | --- | --- | --- |
| **Intensity** | **Examples of Activity** | **Complete Dataset** | **Outlier Removed** |
| Sedentary  Behaviour (SB) | Sitting, sleeping | 0 - 777 | 0 - 792 |
| Light Physical  Activity (LPA) | Casual walking, light  household chores | 778 - 8415 | 793 - 8356 |
| Moderate Physical  Activity (MPA) | Brisk walking, cycling at  moderate pace, jogging | 8416 - 18731 | 8357 - 18437 |
| Vigorous Physical  Activity (VPA) | Running, sprinting | 18732 < | 18438 < |

2. **Then, also to add the obtained cut-points in table 5 (or to create another table) near the values from Rhudy et al. just for a more direct comparison between what is found and what is present in the literature.**

Thank you for highlighting this aspect of the table as it needed further clarification. The table was presented with data from Rhudy for the foot, ankle, and hip, but that study did not have separate data for the shank. Therefore, the shank values in Table 5 are those from the current study. While the text has been updated to clarify this in lines 344-345, it is not overtly clear in the table itself. Therefore, we have adjusted the table caption to clarify this and added information in each line to further highlight this.

**Table 5. Cut-points for MPA and VPA at foot, ankle, and hip wear locations from Rhudy et al [16], with the current study’s cut-points for the shank.**

| **Wear Location** | **MPA cut-point** | **VPA cut-point** |
| --- | --- | --- |
| Foot [16] | 20575 | 22629 |
| Ankle [16] | 14767 | 17818 |
| Shank [current study] | 8357 | 18438 |
| Hip [16] | 4978 | 6227 |

Reviewer #2: I would like to thank the authors for addressing the raised points. Further points below:

Thank you for taking the time to review our paper. Your comments and suggestions have helped enhance the overall quality of the manuscript. Below you will find all comments addressed and incorporated in the updated paper.

1. Concerning the previous raised point 7, it is clear how you performed the LOOCV analysis. But did you split the dataset into a construction set (to train and perform validation, e.g., 80% of the entire dataset) and a test set (e.g., 20% of the entire dataset)? You should show the averaged results from all the iterations of the LOOCV and also the results of the best trained model on the test set. Please update accordingly Figure 2, Table 2 and 3, and further explain how you evaluated the method performances.

If you did not split the entire dataset as explained above, justify it and mention it in the limitations, and clarify what you mean by results from training model and LOOCV analysis.

Thank you for this comment. It highlights that the explanations regarding the training set can be clearer. Additionally, we believe the previous explanation and edits to the methods section may have actually further convoluted the process by overly relying on the association to machine learning. Therefore, we reworked this entire section to help clarify this process entirely. Briefly, it is important to note that a conventional LOOCV in a machine learning model was not used here, but rather we computed cut-points in a manner analogous to this process. In doing so, all accuracy results are fully blind to that individual, and as such the results presented can be taken as a true validation of the model, with no data bleeding from training to validation/test. It is our hope that the new LOOCV section in lines 201-213 helps to more directly convey this message.

2. Concerning the previous raised point 12, please mention the down-sampling at 30 Hz of Axivity data to be able to compare the results with ActiGraph.

Thank you for suggesting this. The down-sampling at 30 Hz of the Axivity data has now been mentioned in lines 178-179.

3. The information of the percentual time spent in different PA intensities is provided by the Bland-Altman plots. Please add in the text the overall averaged reference % of time spent in different PA intensities to provide a clear idea of the different amount of data of the different PA levels. These values can be added in line 327.

These values have now been added to the manuscript to give readers more of an insight of how much activity was completed. On average, participants spent 79.70%, 18.60%, 1.46%, and 0.24% in SB, LPA, MPA, and VPA, respectively. These values were added from lines 315-316, as this information is referenced earlier in the discussion.

4. Please rephrase lines 326-330. Since the activity counts are only based on accelerations, I would not say that the higher VPA cut-point at the shank is related to a higher effort of older adults, but only intensity.

Thank you for highlighting this oversight. This has been modified in the updated document to only include intensity.
